# Edible Flowers in Modern Gastronomy: A Study of Their Volatilomic Fingerprint and Potential Health Benefits

**DOI:** 10.3390/molecules30081799

**Published:** 2025-04-17

**Authors:** Begoña Fernández-Pintor, Rosa Perestelo, Sonia Morante-Zarcero, Isabel Sierra, José S. Câmara

**Affiliations:** 1 Departamento de Tecnología Química e Ambiental, Escuela Superior de Ciencias Experimentales y Tecnología, Universidad Rey Juan Carlos, C/Tulipán s/n, 28933 Móstoles, Madrid, Spain; begona.fernandez@urjc.es (B.F.-P.); sonia.morante@urjc.es (S.M.-Z.); 2 Instituto de Tecnologías para la Sostenibilidad, Universidad Rey Juan Carlos, C/Tulipán s/n, 28933 Móstoles, Madrid, Spain; 3 CQM—Centro de Química da Madeira, Universidade da Madeira, Campus da Penteada, 9020-105 Funchal, Portugal; rmp@staff.uma.pt; 4 Departamento de Química, Facultade de Cièncias Exatas e Engenharia, Universidade da Madeira, Campus da Penteada, 9020-105 Funchal, Portugal

**Keywords:** fresh edible flowers, headspace solid-phase microextraction, volatile compounds, gass chromatography-mass spectrometry, aroma, bioactive compounds

## Abstract

Given the transformation that gastronomy has undergone in recent years, there is a need to characterize some new foods that are being incorporated into the modern diet. Among them, edible flowers stand out, which are used today not only to enhance the organoleptic properties of gourmet dishes but also for some of the beneficial properties they provide to human health. In this study, the volatilomic fingerprint of seven edible flowers that are used daily in Michelin-starred restaurants on Madeira Island was established. For this purpose, the extraction of volatile organic metabolites (VOMs) was carried out using the headspace solid-phase microextraction (HS-SPME) technique followed by gas chromatography coupled to mass spectrometry (GC-MS). The results showed a wide variability among the analyzed flowers. While fewer VOMs were detected in some flowers, other flowers, such as *Viola tricolor* and *Rosa* spp., exhibited a greater number of these compounds. *Acmella oleracea* had the highest number of detected VOMs. Each of these VOMs contributes to the characteristic aroma representative of the respective flower, highlighting their potential health benefits, as some are known for their anti-inflammatory, antimicrobial, and even anticancer properties.

## 1. Introduction

In recent years, gastronomy has undergone a significant transformation that involves the use of new ingredients beyond those conventionally used. One of the most notable and visually appealing elements that has gained popularity is the use of edible flowers. These not only enhance the aesthetic appeal of dishes but also offer a variety of flavors and textures that enrich the culinary experience [1]. Incorporating flowers into the diet, or floriphagia, is not a novel concept; ancient civilizations such as the Romans and the Chinese utilized flowers in their recipes [2,3]. However, in modern gastronomy, their use has been rediscovered and reinvented by creative and innovative chefs aiming to surprise their customers. Numerous edible flowers exist, each with unique characteristics. Among the most popular are marigolds, roses, and squash blossoms. These flowers can be utilized in several culinary applications, ranging from salads and soups to desserts and beverages [4]. For instance, lavender flowers are renowned for their ability to flavor desserts, while marigold flowers can provide spicy notes to salads. In addition to their visual and gustatory appeal, many culinary flowers possess beneficial nutritional properties. Some of them contain antioxidant compounds, vitamins, and minerals that contribute to a balanced diet [5,6]. For example, hibiscus flowers are rich in vitamin C and antioxidants, making them a healthy option for infusions and desserts [7]. However, it is crucial to be aware that not all flowers are safe for consumption. While many edible flowers offer health benefits, others may have potentially harmful compounds in their composition, causing allergic, among other, reactions. For instance, some flowers contain alkaloids, which can be harmful if consumed in large quantities. Therefore, it is important to ensure that the flowers used in gastronomy are properly identified and sourced from safe, non-toxic varieties. Knowledge of the potential risks associated with certain flowers is essential for both chefs and consumers to avoid any adverse effects [6].

The trend of using flowers in gastronomy has been driven by the sustainable cooking movement and the pursuit of local and organic ingredients [8]. High-end chefs and gourmet restaurants have embraced this practice, creating dishes that are not only delicious but also visually striking. Furthermore, the popularity of social media has played a crucial role, as dishes adorned with flowers are highly photogenic and attractive for sharing on social platforms [9]. The introduction of flowers in modern gastronomy represents a fusion of tradition and creativity. This approach not only enriches the available flavors but also elevates the presentation of dishes to an art form.

Volatile organic metabolites (VOMs) found in most edible flowers play a crucial role in modern gastronomy for the flavor and aroma nuances they provide. These compounds are responsible for the unique fragrances that each flower may offer, and their presence can transform a common recipe into an extraordinary sensory experience. Flowers emit a variety of VOMs, including terpenes, phenols, esters, aldehydes, and ketones [10]. These compounds are responsible for the characteristic aromas of each flower. For example, terpenes are common in flowers such as roses and lavender, providing sweet and floral notes [11]. In nature, VOMs serve several functions. They attract pollinators, such as bees and butterflies, facilitating plant reproduction. Additionally, some VOMs can act as defense mechanisms, repelling herbivores or attracting their predators [12]. In culinary applications, the aromas of flowers are used to add depth and complexity to dishes. Jasmine flowers, for example, are known for their sweet and exotic aroma, which is used in teas and desserts. Orange blossom flowers, with their citrus fragrance, are popular in baking and beverage preparation. The incorporation of culinary flowers in modern gastronomy not only enriches dishes with vibrant colors and textures but also introduces an olfactory dimension that can transform the culinary experience [13]. The VOMs and aromas of flowers are a powerful tool for chefs seeking to innovate and surprise their diners.

Extraction and analysis of VOMs from edible flowers is crucial for understanding their aromatic properties and potential applications. Several extraction methods are used to obtain VOMs from flowers: supercritical fluid extraction [14], microwave-assisted extraction [15], headspace solid-phase microextraction (HS-SPME), among others. HS-SPME has been used in many studies because of its versatility and simplicity [16,17,18]. This technique, based on a coated fiber, is a rapid, miniaturized method that uses minimal amounts of solvents and reagents and is mainly used in the analysis of VOMs in foods to obtain their volatile fingerprint [19,20,21]. Gas chromatography is the gold standard analytical technique in the research of VOMs. It is used to separate, identify, and quantify the extracted VOMs. When combined with mass spectrometry (GC-MS), it allows for precise identification of the compounds present in the samples. Specifically, reviewing the literature related to the analysis of VOMs in edible flowers, there are some studies that apply HS-SPME/GC-MS. For example, Giannetti et al. (2024), used this methodology to determine the aromatic profile of 85 samples of four edible flower cultivars [1]. Marchioni et al. (2020) utilized this technique to study the effect of post-harvest treatment on the nutraceutical and aromatic properties of various culinary flowers, and also conducted a study to determine the aromatic profile of flowers from the Lamiaceae family [22,23]. Najar et al. (2019) employed HS-SPME/GC-MS to identify the VOMs in culinary flowers from the Agastache genus [24]. Fernandes et al. (2019) used this technique to determine the bioactive compounds of five culinary flowers commonly used in recent years [25]. Izcara et al. (2022) also analyzed some edible flowers in order to characterize their volatile fingerprint using HS-SPME/GC-MS [19].

The objective of this study was to establish the volatilomic fingerprint of seven fresh edible flowers commonly used in gourmet gastronomy (*Antirrhinum majus*, Plantaginaceae family; *Begonia* spp., Begoniaceae family; *Borago officinalis*, Boraginaceae family; *Rosa* spp., Rosaceae family; *Acmella oleracea*, Asteraceae family; *Lobularia maritima*, Brassicaceae family; *Viola tricolor*, Violaceae family) through the application of HS-SPME/GC-MS methodology to isolate and identify the VOMs responsible for the sensory characteristics of each flower. This study represents a significant contribution to the field of volatilomics as applied to gourmet cuisine by characterizing the volatile compound profiles of edible flowers used in Michelin-starred restaurants in Madeira, including underexplored species such as *Acmella oleracea* and *Lobularia maritima*. Furthermore, the study aims to investigate the possible health benefits associated with the presence of these VOMs. These findings not only enhance the scientific understanding of the volatile profiles of these flowers but also provide a foundation for practical applications in ingredient selection for gourmet cuisine, supporting the creation of dishes that optimize both sensory experiences and health benefits.

## 2. Results

### 2.1. Volatilomic Fingerprinting from Edible Flowers

**Figure 1:**
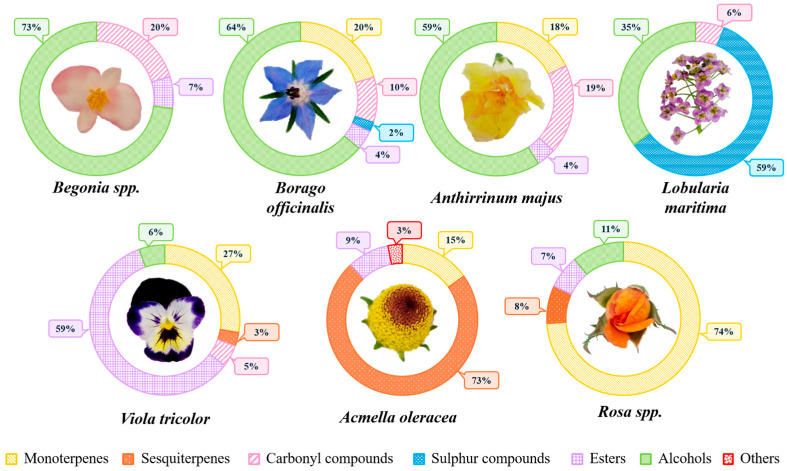
Distribution of chemical families of VOMs identified in the analyzed edible flowers.

After performing the HS-SPME extraction and subsequent GC-MS analysis, chromatograms were obtained for each of the analyzed edible flowers (Figure S1). Following the interpretation of these chromatograms, a total of 102 VOMs, including 17 carbonyl compounds, three sulfur compounds, 15 esters, one furanic compound, 20 monoterpenoids, 16 alcohols, and 30 sesquiterpenoids, were identified. The detailed list of all VOMs identified in the analyzed culinary flowers and their respective obtained data, including retention times, KIs, molecular formulas, chemical families, and relative peak areas, is shown in Table 1, while the percentage of relative area corresponding to each chemical family obtained for each of the analyzed edible flowers is shown in Figure 1.

#### 2.1.1. *Begonia* spp.

As shown in Table 1, the volatilomic fingerprint of this flower is quite limited. Only four VOMs were tentatively identified, with two of them being alcohols (73%), as shown Figure 1. Additionally, one carbonyl compound (20%) and one ester (7%) were detected. As can be seen in Table S1, this is the flower that presented the lowest number of VOMs, as well as the smallest total amount in terms of total relative area.

#### 2.1.2. *Borago officinalis*

In the case of this flower, Table 1 and Figure 1 show that 20 VOMs were tentatively identified, with alcohols being the most prominent in the sample (64%), followed by monoterpenes (20%) and carbonyl compounds (10%). Additionally, esters and sulfur compounds were identified in proportions of 4% and 2%, respectively. Of the identified monoterpenes eucalyptol (23) and p-cymene (32), eucalyptol stands out due to its relative peak area (19.9%). Eucalyptol has a characteristic minty aroma and is well-known for its anti-inflammatory properties, particularly in the treatment of respiratory issues. Additionally, it may exhibit antimicrobial properties. Therefore, a high percentage of this compound in the flower suggests that its consumption could be beneficial for human health.

#### 2.1.3. *Anthirrinum majus*

In this flower, 22 VOMs were tentatively identified, as shown in Table 1, with alcohols (59%) being the most dominant chemical group (Figure 1). This was followed by carbonyl compounds (19%), monoterpenes (18%), and finally, esters (4%). Among the monoterpenes, seven of the present VOMs stand out for their potential bioactivity: *trans*-β-ocimene (29) at 7%; limonene (22) at 2.5%; β-pinene (14) at 2.4%; β-myrcene (19) at 2%; α-pinene (10) at 1.8%; 3-carene (17) at 1.2%; and eucalyptol (23) at 1%. Most of these VOMs have demonstrated beneficial properties, which will be explained in detail in subsequent sections.

#### 2.1.4. *Lobularia maritima*

Six VOMs were tentatively identified in *Lobularia maritima* (Table 1), with a significant presence of sulfur compounds (59%) and alcohols (35%), as shown in Figure 1. Notable sulfur compounds include 3-butenyl isothiocyanate (51) and allyl isothiocyanate (40), while prominent alcohols are phenylethyl alcohol (97) and (E)-2-hexen-1-ol (44). The minority group consists of carbonyl compounds, representing only 6% of the total area percentage of the sample, within which acetaldehyde (1) and hexanal (13) were detected. Despite finding a limited number of VOCs in this flower, the total amount of VOMs in terms of relative area is 44, as shown in Table S1.

#### 2.1.5. *Acmella oleracea*

This flower stood out due to the presence of numerous sesquiterpenes, as this family represented 73% of the total area of the compounds identified in this sample (Figure 1). Additionally, it is the sample in which were identified the highest number of VOMs (50) (Table 1). Additionally, it is the flower that presented the highest amount of VOMs, with a total relative area of 3782 (Table S1). The compound with the largest total area was β-caryophyllene (70), accounting for 42% of the total volatilomic fraction. This was followed by β-bisabolene (84) at 11%, α-caryophyllene (78) at 4%, and δ-amorphene (88) at 2%. Furthermore, 22 other sesquiterpenes were detected in smaller proportions: α-cubebene (53), δ-elemene (55), cyclosativene (57), copaene (59), β-bourbonene (60), cyperene (66), β-cubebene (67), aromandrene (71), δ-cadinene (73), humelene (74), β-farnesene (77), α-muurolene (80), germacrene D (81), bicyclogermacrene (86), α-farnesene (87), δ-selinene (91), β-muurolene (94), β-amorphene (96), α-calacorene (98), caryophyllene oxide (99), nerolidol (100), t-cardinol (101), and caryophylladienol I (102).

Monoterpenes accounted for 15% of the total volatilomic fraction, followed by esters (9%), and other compounds (3%). Among the monoterpenes, notable compounds include 3-carene (17) at 7%, β-phellandrene (25) at 3%, and geraniol (95) at 1.7%. Additionally, 13 other monoterpenes were found in smaller proportions: α-pinene (10), sabinene (11), camphene (12), β-pinene (14), α-phellandrene (15), β-myrcene (19), limonene (22), γ-terpinene (28), *trans*-β-ocimene (29), p-cymene (32), α-terpinolene (33), *trans*-alloocimene (43), and fenchone (46).

#### 2.1.6. *Viola tricolor*

In this flower, 38 VOMs were found, as shown in Table 1, with esters being the most representative group (59%), followed by monoterpenes (28%), and in smaller proportions, alcohols (6%), carbonyl compounds (5%), and sesquiterpenes (3%) (Figure 1). Among the esters, methyl salicylate (89) stands out, contributing 43%. For monoterpenes, *trans*-β-ocimene (29) is notable at 25%, followed by seven other VOMs in smaller proportions: α-pinene (10), camphene (12), β-pinene (14), β-myrcene (19), limonene (22), eucalyptol (23), and fenchone (46). Regarding sesquiterpenes, six were detected: β-bourbonene (60), β-caryophyllene (70), germacrene D (81), α-citral (82), β-bisabolene (84), and α-farnesene (87).

#### 2.1.7. *Rosa* spp.

As shown in Table 1, 33 VOMs were tentatively identified, with monoterpenes (74%) being the most prominent chemical family (Figure 1). The major compound is geraniol (95), accounting for 69% of the total volatilomic fraction. Other identified monoterpenes include β-myrcene (19), limonene (22), eucalyptol (23), β-phellandrene (25), *trans*-β-ocimene (29), citronellal (52), β-cyclocitral (76), α-caryophyllene (78), and γ-muurolene (90). Additionally, 11% of the VOMs were alcohols, with phenylethyl alcohol (97) being notable. Sesquiterpenes accounted for 8% of the total volatilomic fingerprint, with α-citral (82) standing out, and esters made up 7%, with 2-phenylethyl acetate (92) being remarkable. Other sesquiterpenes found include copaene (59), theaspirane B (64), β-caryophyllene (70), and β-bisabolene (84). This flower is the second highest in terms of VOM quantity, with a total relative area of 905.

### 2.2. Odor of Some Identified VOMs and Their Potential Bioactive Effects

**Figure 2:**
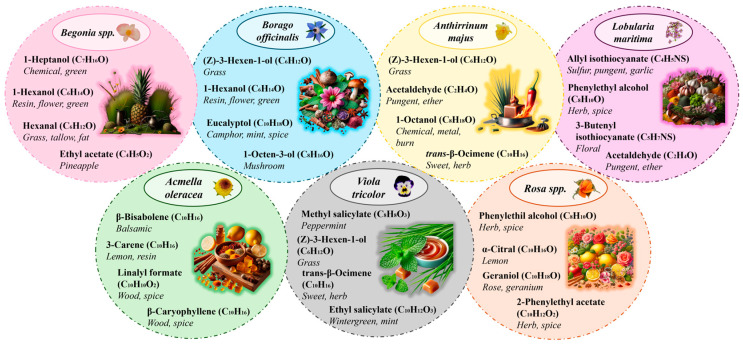
Characteristic odors and flavors related with the majors VOMs identified in the analyzed edible flowers (Flavornet).

Figure 2 shows the major VOMs identified in each of the analyzed flowers, as well as the characteristic aroma of each compound (Flavornet). Additionally, Table 2 presents the beneficial effects found in the literature for some of the VOMs detected in the analyzed flowers.

#### 2.2.1. *Begonia* spp.

This flower is not very aromatic, as it only presents four VOMs identified using HS-SPME/GC-MS. The major ones are 1-heptanol, 1-hexanol, and hexanal, which primarily provide grassy aromas but also contribute notes of resin, flower, chemical, tallow, and fat. On the other hand, ethyl acetate provides a pineapple note, although its relative area is the lowest of the four detected compounds. One of the culinary uses of this flower is its use as decoration in numerous dishes, with its use in salads standing out. Another use is the preparation of sauces and side dishes based on this flower to accompany different types of meats. Additionally, it can be used to make desserts such as ice creams or granitas, as its acidic flavor provides a contrasting sensation to the sweet taste typically found in these foods.

Regarding bioactive properties, 1-heptanol and 1-hexanol provide antifungal properties, as demonstrated in the study by Li et al. (2022) [29]. Additionally, hexanal provides antimicrobial properties against pathogenic microorganisms [30], and ethyl acetate has also antimicrobial properties [31].

#### 2.2.2. *Borago officinalis*

In the case of *Borago officinalis*, the major compound is (Z)-3-hexen-1-ol (42), which is characterized by a grassy smell, followed by eucalyptol (23), which provides mint, spice, and camphor notes. Another major compound is 1-hexanol (39), whose predominant aromas are resin, floral, and green, and finally, 1-octen-3-ol (48), which is characterized by a mushroom aroma. These flowers are characterized by their distinctive bright blue color, making one of their main uses as a decorative element that enhances the appearance of dishes such as salads or soups. On the other hand, they are widely used in beverages, such as cocktails or infusions. Another of their uses is freezing them in ice cubes, which gives drinks a unique visual touch.

One of the potentially bioactive compounds detected in this flower is eucalyptol, which is known for its anti-inflammatory, antioxidative, antihyperglycemic, antimicrobial, antihypertensive, anti-tumoral, antinociceptive, antipyretic, and analgesic properties [32]. Another major VOM found in this flower is 1-octen-3-ol, which imparts antioxidant activity [33]. p-Cymene has many biological activities, such as antibacterial, antifungal, antiviral, anti-inflammatory, antidiabetic, antioxidant, and analgesic. It is also antitumoral, anticancer, and a neuroprotective agent [34].

#### 2.2.3. *Anthirrinum majus*

In the case of this flower, the major VOM is 1-octanol (65), followed by (Z)-3-hexen-1-ol (42), acetaldehyde (1), and *trans*-β-ocimene (29). These VOMs give the flower characteristic olfactory notes, including grassy, sweet yet spicy aromas, and other notes such as chemical, metallic, or burnt. The main use of these flowers is for decoration, due to the variety of colors they present, making them ideal for decorating pastries, including wedding cakes and other desserts. Occasionally, they are used in the same way as *Borago officinalis*, by freezing them in ice cubes for cocktails. These flowers do not stand out much for their flavor, but rather for their visual appearance.

One of the major compounds, 1-octanol, provides antifungal properties [35]. However, this flower also contains other compounds with potentially bioactive properties in smaller proportions. α-Pinene exhibits antidiabetic, antifungal, antioxidant, antiproliferative, antitumor, and cytotoxic properties [36,37]; β-Pinene demonstrates antitumor, anti-inflammatory, antimicrobial, antioxidant, antineoplastic, and chemoprotective properties [38,39,40]. 3-Carene provides antimicrobial, antioxidant, and anticancer properties [41]; β-Myrcene has been shown to be analgesic, anti-inflammatory, antibiotic, anticancer, and antioxidant [42,43]. Limonene exhibits antimutagenic, antitumor, antioxidant, antimicrobial, antiproliferative, and chemoprotective properties [38,40,44], as well as eucalyptol, which has already been described in *Borago officinalis*.

#### 2.2.4. *Lobularia maritima*

The major VOM is a sulfur compound, 3-butenyl isothiocyanate (51), characterized by its floral aroma, followed by phenylethyl alcohol (97) with spicy and grassy aromas. Lastly, allyl isothiocyanate (40) and acetaldehyde (1) provide sulfurous, spicy, and even garlic-like aromas. In gastronomy, it is commonly used for decorating both sweet and savory dishes. In savory foods, its use in salads stands out, as it provides a light floral aroma that makes the culinary experience unique. This aroma is also utilized in beverages, such as cocktails. On the other hand, one of its main uses is in decorating desserts, like cakes or mousses, as it is a small and delicate flower that adds a visually elegant touch.

The compound 3-butenyl isothiocyanate has demonstrated cytotoxic capacity, as it induces cytotoxicity in cancer cells, thus proving its anticancer potential [45]. Phenylethyl alcohol is a substance with antimicrobial, antioxidant, and antienzymatic effects, as it inhibits the activity of the enzyme tyrosinase [46]. On the other hand, allyl isothiocyanate is a compound with anticancer, antibacterial, antifungal, anti-inflammatory, and antioxidant properties [47].

#### 2.2.5. *Lobularia maritima*

This flower has a clear presence of β-caryophyllene (70) and linalyl formate (69), which provide woody and spicy aromas. Additionally, there is a notable presence of β-bisabolene (84), which gives a balsamic aroma, and 3-carene (17), which adds lemon and resin notes. These flowers are added to culinary dishes primarily for the sensation they produce when eaten. They are flowers that cause an electric feeling and a numbing sensation on the tongue, making the diner’s experience truly unique. For this reason, they are used in salads, carpaccios, ceviches, and other savory dishes. They are also widely used in the beverage realm, both as decoration and to alter the perception of the drink’s flavors.

The major compound of this flower is β-caryophyllene, which has demonstrated anticancer and analgesic properties [48]. On the other hand, β-bisabolene exhibits cytotoxic, anti-inflammatory, and antibacterial properties [49], while 3-carene, already discussed in the section on *Antirrhinum majus*, also has noteworthy properties. Other potentially bioactive compounds, such as α-pinene, β-pinene, β-myrcene, limonene, and p-cymene, have been previously explained for their bioactive properties.

Furthermore, sabinene exhibits antifungal and antimicrobial properties [50]; camphene shows antimicrobial and antioxidant activities [51,52]; γ-terpinene has anti-inflammatory and antioxidant effects [38,53]; and α-terpinolene is an antioxidant [54]. Geraniol demonstrates chemopreventive, antimutagenic, and anti-inflammatory activities [55,56]. Caryophyllene oxide exhibits antibacterial, antioxidant, antiproliferative, cytotoxic, and analgesic properties [57], whereas β-phellandrene possesses antibacterial, antifungal, anticancer, antidiabetic, antioxidant, analgesic, antiviral, and anti-inflammatory properties [58,59].

**Figure 3:**
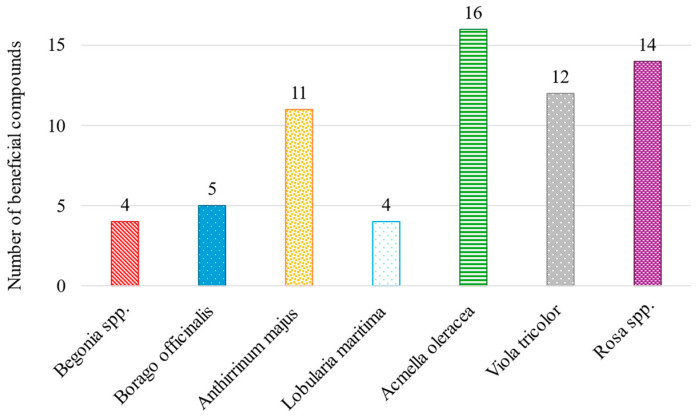
Number of VOMs with potential bioactive health effects identified in the investigated edible flowers.

*Acmella oleracea* is the flower with the highest number of potentially beneficial compounds, followed by *Rosa* spp. and *Viola tricolor*, as shown in Figure 3. This highlights the potential of this flower, as it would not only enhance the visual appeal of gastronomic dishes but also contribute to improvements in the health of its consumers.

#### 2.2.6. *Viola tricolor*

This flower contains a major compound, methyl salicylate (89), as well as ethyl salicylate (93), which provides mint aromas. Additionally, *trans*-β-ocimene (29) and (Z)-3-hexen-1-ol (42) provide grassy aromas and some sweet notes. These flowers add visual appeal to dishes thanks to their colors, which range from yellow to purple. They are used in salads as a decorative element, but their main use is in pastry, where they are employed to decorate cakes, cookies, and pastries. Another way to prepare them is by crystallizing them with sugar, making them an ideal garnish for desserts.

Regarding the potentially bioactive properties, the major compound, methyl salicylate, exhibits significant properties, including anti-inflammatory, analgesic, antipyretic, and antifungal effects, while also presenting fewer gastrointestinal side effects compared to other compounds such as aspirin [60]. Additionally, it contains other previously mentioned compounds in smaller proportions: α-pinene, β-pinene, β-myrcene, limonene, camphene, eucalyptol, and β-caryophyllene.

#### 2.2.7. *Rosa* spp.

In the case of the rose, the most abundant VOM is geraniol (95), which provides rose and geranium aromas. Additionally, both phenylethyl alcohol (97) and 2-phenylethyl acetate (92) contribute herbal and spicy aromas. Lastly, the presence of α-citral (82) adds a lemon aroma to this sample. These flowers have been used since ancient times for both their flavor and visual appeal. They are used in sweet dishes for decoration and flavoring, with the petals commonly utilized in various forms: fresh, dehydrated, or crystallized. One of their main uses is the preparation of rose water, which enhances the flavor of desserts like puddings, especially in South Asia. In beverages, they are also widely used, being added to cocktails, infusions, teas, and other drinks like wines or liqueurs. Although their use in savory dishes is less common, they can also be incorporated to add color and sweet flavors to foods such as cheeses or sauces.

This sample contains several of the previously described compounds with bioactive properties, such as geraniol, phenylethyl alcohol, β-myrcene, limonene, eucalyptol, β-phellandrene, and β-caryophyllene. Additionally, this flower features a compound unique to it, γ-muurolene, which is characterized by its anti-inflammatory, antimicrobial, antioxidant, and cytotoxic properties [61].

Overall, edible flowers not only improve the aesthetic quality of gourmet dishes but also contribute significant nutritional value. *Antirrhinum majus* and *Lobularia maritima*, for instance, contains bioactive compounds that can contribute to reducing oxidative stress or chronic inflammation [62,63]. *Borago officinalis* and *Rosa* spp. contribute to the nutritional enhancement, being are very rich in essential vitamins, antioxidants, and fatty acids, promoting overall health [64], whereas other compounds contribute to several flavors ranging from tangy (*Begonia* spp.) to numbing (*Acmella oleracea*), adding complexity to gourmet meals while providing therapeutic benefits. Therefore, the incorporation of edible flowers into gastronomy enhances both the sensory and nutritional profile of dishes. Their bioactive compounds offer anti-inflammatory, antioxidant, hepatoprotective, and immune-supporting properties that align with modern dietary trends focused on wellness.

### 2.3. Multivariate Statistical Analysis

**Figure 4:**
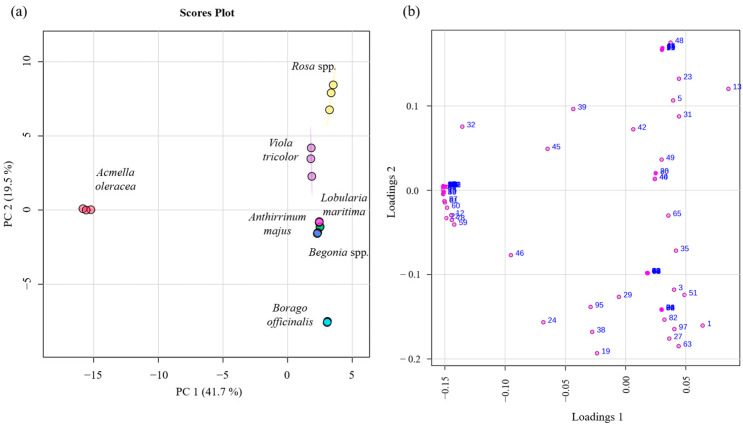
Statistical analysis using PCA score plot (**a**) and loading plot (**b**) of the VOMs identified in the investigated edible flowers. The numbers correspond to the VOMs (see Table 1).

**Figure 5:**
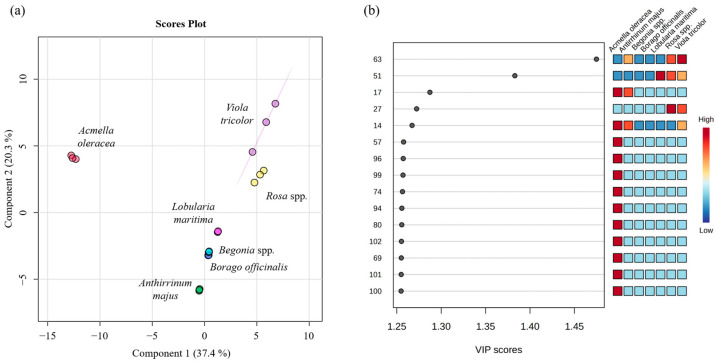
Statistical analysis using PLS-DA score plot (**a**) and variable importance in the projection (VIPs) plot (**b**) of the VOMs identified in the analyzed edible flowers. The numbers correspond to the VOMs (see Table 1).

A statistical analysis was carried out using the MetaboAnalyst 6.0 program to obtain the principal component analysis (PCA) and partial least squares-discriminant analysis (PLS-DA) as described in Section 3.5. Figure 4 and Figure 5 show the PCA score plot and PCA loading plot, which visually display the differences and similarities between the volatilomic fingerprint of the edible flowers analyzed, as well as the distribution of VOMs in the four quadrants of the graph. The variance of the first principal component (PC1) was 41.7%, while the second (PC2) was 19.5%, so the total of both components was 61.2% of the data variability, indicating differences between the edible flowers analyzed. A cluster can be seen among *Begonia* spp., *Anthirrinum majus*, and *Lobularia maritima*, indicating that these flowers have the most similar volatilomic fingerprints.

All samples analyzed, except *Acmella oleracea*, are in the positive PC1. In the case of PC2, *Rosa* spp. and *Viola tricolor* are in the positive quadrant, while *Borago officinalis*, *Lobularia maritima*, *Anthirrinum majus*, and *Begonia* spp. are in the negative. *Acmella oleracea* is located right in the middle of both quadrants. The positive PC1 and PC2 quadrant is characterized by the presence of VOMs such as 3-methyl-butanal (5), hexanal (13), limonene (22), eucalyptol (23), 3-methyl-1-butanol (26), 3-octanone (31), allyl isothiocyanate (40), (Z)-3-hexen-1-ol (42), (E)-2-hexen-1-ol (44), 1-octen-3-ol (48), 1-heptanol (49), and α-muurolene (80). The negative PC1 and PC2 quadrant includes β-myrcene (19), (Z)-2-hexenal (24), *trans*-β-ocimene (29), 6-methyl-5-hepten-2-one (38), fenchone (46), copaene (59), α-caryophyllene (78), and geraniol (95). Positive PC1 and negative PC2 include acetaldehyde (1), ethyl acetate (3), ethyl hexanoate (27), 3-hexen-1-ol acetate (35), 3-butenyl isothiocyanate (51), ethyl nonanoate (63), 1-octanol (65), α-citral (82), and phenyethyl alcohol (97). Finally, negative PC1 and positive PC2 include p-cymene (32), 1-hexanol (39), and (E)-4-hexen-1-ol (45), among others.

A total variance of 57.7% was obtained by the first two principal components obtained from PLS-DA (37.4% of the PC1 and 20.3% of the PC2). Furthermore, 15 differently expressed VOMs were found with presented VIP scores ≥ 1.25, being the most relevant VOMs and having the greatest discriminatory power to characterize the seven culinary flowers studied. Among these 15 significant VOMs, the majority were sesquiterpenes: cyclosativene (57), humelene (74), α-muurolene (80), β-muurolene (94), β-amorphene (96), caryophyllene oxide (99), neronidol (100), t-cadinol (101), and cariophylladienol I (102). These were followed by esters: ethyl hexanoate (27), ethyl nonanoate (63), and linalyl formate (69); monoterpenes; β-pinene (14), and 3-carene (17); and, finally, one sulfur compound, 3-butenyl isothiocyanate (51).

**Figure 6:**
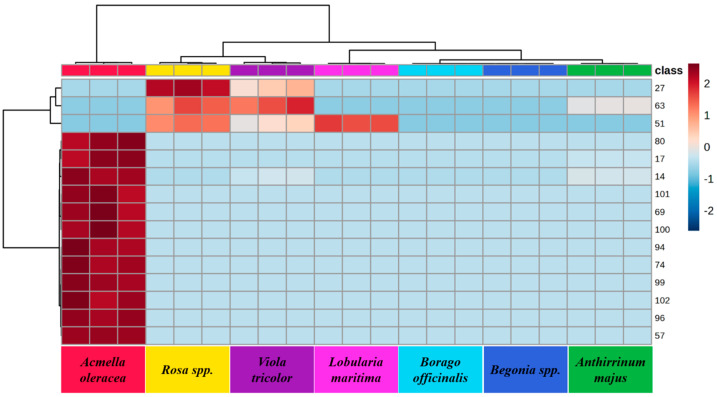
HCA using the 15 significant VOMs identified in the edible flowers analyzed, obtained by ANOVA. The color of the graphic ranges from dark red, which indicates high correlation of VOMs with the corresponding flower, to light blue, which indicates low association of VOMs to the target flower. The dendrogram associated was generated with Ward’s algorithm and Euclidean distance analysis. The numbers correspond to the VOMs (see Table 1). These VOMs are: ethyl hexanoate (27); ethyl nonanoate (63); 3-butenyl isothiocyanate (51); α-muurolene (80); 3-carene (17); β-pinene (14); t-cardinol (101); linalyl formate (69); neronidol (100); β-muurolene (94); humelene (74); caryophyllene oxide (99); cariophylladienol I (102); β-amorphene (96); cyclosativene (57).

The *p* values obtained by one-way ANOVA with Fisher post-hoc test (*p* < 0.05) indicated that the 102 VOMs identified were significantly different among the investigated culinary flowers. Moreover, hierarchical cluster analysis (HCA) was also performed using the 15 most significant VOMs identified in culinary flower samples obtained by ANOVA. The resulting dendrogram associated with the heat map was performed by Euclidean distance through Ward’s clustering method (Figure 6), providing intuitive visualization of the data set, which, along with the statistical analyses carried out previously, allows better identification of the inherent clustering patterns between each culinary flower. It can be observed that most of the VIPs are concentrated in *Acmella oleracea*, confirming the broad volatilomic fingerprinting of this culinary flower.

## 3. Materials and Methods

### 3.1. Chemical and Reagents

All used reagents were of analytical grade. The 3-octanol (internal standard, IS) and sodium chloride (NaCl, 99.5%) were purchased from Sigma Aldrich (Madrid, Spain). The alkane series (C_8_ to C_20_) used to determine the KI, prepared in a concentration of 40 mg/L in n-hexane, were acquired from Fluka (Buchs, Switzerland). Ultrapure water was obtained using a Milli-Q^®^ system (Millipore, Bedford, MA, USA). The SPME fiber coated with divinylbenzene/carboxen/polydimethylsiloxane (DVB/CAR/PDMS) (50/30 μm), the SPME holder for manual sampling, and the amber glass vials were purchased from Supelco (Bellefonte, PA, USA).

### 3.2. Fresh Edible Flowers

Before conducting the study, a preliminary investigation was carried out regarding the flowers used in gourmet cuisine dishes served in Michelin-starred restaurants. For this purpose, consultations were made with two Michelin-starred restaurants on Madeira Island, Portugal. Seven of the most edible flowers used were selected for volatilomic fingerprint analysis. These flowers were: *Antirrhinum majus* (snapdragon, Scrophulariaceae family), *Begonia* spp. (begonia, Begoniaceae family), *Borago officinalis* (borage, Boraginaceae family), *Rosa* spp. (rose, Rosaceae family), *Acmella oleracea* (toothache plant, Asteraceae family), *Lobularia maritima* (sweet alyssum, Brassicaceae family), and *Viola tricolor* (wild pansy, Violaceae family).

All of them were purchased from a supermarket on the Madeira Island, where they are commercially available to the public, and were cultivated in the region by a local company. The flowers were collected in June 2025. The samples were acquired on the same day of their analysis and, as indicated by the manufacturer, the fresh flowers were stored under refrigeration (4 °C) until the time of analysis. Finally, before each extraction, the samples were homogenized to ensure uniform size and a representative analysis.

### 3.3. HS-SPME Procedure

**Figure 7:**
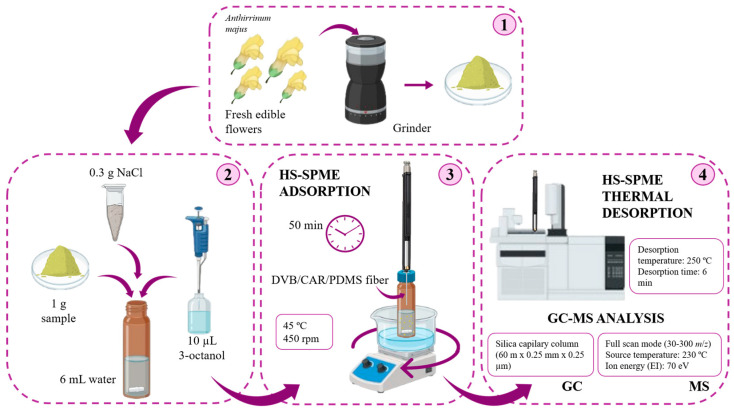
Workflow of the analytical methodology followed for the VOMs determination in edible flowers.

With slight modifications, the HS-SPME procedure was adapted from the conditions described by Izcara et al. (2022) [19]. Prior to use, each day, the SPME fiber was thermally conditioned for 10 min according to the manufacturer’s instructions to verify the absence of carryover analytes. For the extraction of VOMs from culinary flowers, 1 g of each sample, 6 mL of ultrapure water, and 0.3 g of NaCl were placed into a 50 mL amber glass vial containing a stirring microbar (2 × 0.5 mm, 450 rpm). Before sealing the vial with a PTFE-faced silicone septum, 10 μL of 3-octanol, as internal standard (100 μg/mL), was added. The vial was placed in a thermostatic block at 45 ± 1 °C. HS-SPME extractions were performed by exposing the SPME fiber (DVB/CAR/PDMS) to the headspace sample vial for 50 min to absorb VOMs in the fiber. Finally, the fiber was retracted into the holder needle, removed from the vial, and placed into the GC-MS injector at 250 °C for 6 min for thermal desorption of the target analytes (see Figure 7). All analyses were conducted in triplicate (n = 3).

### 3.4. GC-qMS Analysis

Chromatographic separations of VOMs from culinary flowers were conducted using an Agilent Technologies 6890N (Palo Alto, CA, USA) gas chromatography system equipped with a SUPELCOWAX^®^ 10 fused silica capillary column (60 m × 0.25 mm i.d. × 0.25 μm film thickness) provided by Supelco (Bellefonte, PA, USA). Helium (Helium N60, Air Liquid, Sines, Portugal) was used as the carrier gas at a flow rate of 1 mL/min and column-head pressure of 13 psi. The temperature program was set as follows: 40 °C for 1 min, increased to 220 °C with a ramp of 2.5 °C/min, and finally held isothermally at 220 °C for 10 min. MS detection was carried out using an Agilent 5975 quadrupole inert mass selective detector in full scan mode (FS). The temperatures of the injector and quadrupole mass spectrometry detector (q-MS) were 250 °C and 230 °C, respectively. The injection was performed with a splitless injector using a 0.75 mm i.d. insert. Ion energy for electron impact ionization (EI) was 70 eV. The electron multiplier was adjusted according to the autotune procedure. The mass range acquired was 30–300 *m*/*z*. The VOMs were tentatively identified comparing the RTs, the KI and the mass spectra with standards and the mass spectra with the National Institute of Standards and Technology (NIST) MS 05 spectral database (Gaithersburg, MD, USA). The KI values were obtained using the van Den Dool and Kratz (1963) equation. An n-alkanes solution from C_8_ to C_20_ series was employed to determine the KI, and the values were compared, when available, to those reported in the literature for similar columns [26,27,28] and online databases (the Pherobase and Flavornet).

### 3.5. Statistical Analysis and Data Treatment

The statistical analysis of the data was conducted using the MetaboAnalyst 6.0 web-based tool [65,66]. The raw GC-qMS data were pre-processed to eliminate VOMs with miss values and subsequently normalized (data transformation by cubic root and data scaling by autoscaling). The dataset was then subjected to a one-way analysis of variance (ANOVA) followed by Fisher’s test for post-hoc multiple comparisons of means from seven edible flower varieties at a *p*-value < 0.05 to identify significant differences. PCA and PLS-DA were employed to provide insights into the separations among the edible flowers under study and to detect VOMs that may represent differences among the sample sets. PLS-DA can identify VOM sets that best discriminate among the different edible flowers by eliminating redundant variables and reducing the size of the data matrix. VOMs with a VIP score of 1.25 or higher, and those differentially expressed in univariate analysis, were considered potential markers for characterizing edible flower varieties. HCA was conducted using the 15 most significant VOMs identified in culinary flower samples through ANOVA. This analysis was performed using the Ward’s algorithm and Euclidean distance, aiming to identify clustering patterns for the characterization of the culinary flowers analyzed.

## 4. Conclusions

In this work, the volatilomic fingerprint of seven edible flowers commonly used in various gourmet cuisine restaurants on Madeira Island has been characterized. After performing an extraction using HS-SPME and subsequent analysis by GC-MS, 102 VOMs were identified. Most of the analyzed flowers contained a large number of VOMs, with *Acmella oleracea* standing out as the flower with the most complex volatilomic fingerprint. Moreover, scientific evidence suggests that many of these VOMs have beneficial properties for human health, demonstrating that edible flowers are not only new elements added to dishes for their visual appeal but also that they possess potential health benefits. The seven flower species analyzed contained several compounds, such as α-pinene, β-myrcene, and eucalyptol, with antibacterial, antioxidant, and anti-inflammatory properties, among others. Additionally, the different odors and flavors that VOMs contribute to each flower have been identified, making each one unique and giving it a characteristic aroma, thereby enhancing the culinary experience for consumers. These results are promising, as they highlight the beneficial properties of these novel foods and suggest the need for further research in the future to explore their potential applications in nutrition and health.

## Figures and Tables

**Table 1:** Volatile composition of *Begonia* spp., *Borago officinalis*, *Anthirrinum majus*, *Lobularia maritima*, *Acmella oleracea*, *Viola tricolor*, and *Rosa* spp. and their respective retention time (RT), theoretical and experimental Kovat’s index values, and relative area as determined by HS-SPME/GC-MS.

Peak nº	RT ^a^ (min)	KI_calc_^b^	KI_lit_^c^	VOMs	MF ^d^	Chemical Family	Relative Area ^e^ ± SD						
							*Begonia* spp.	*Borago officinalis*	*Anthirrinum majus*	*Lobularia maritima*	*Acmella oleracea*	*Viola tricolor*	*Rosa* spp.
1	6.03	746	748	Acetaldehyde	C_2_H_4_O	CC	-	-	1.03 ± 0.03	2.16 ± 0.22	-	0.34 ± 0.02	1.12 ± 0.13
2	6.51	803	-	Dimethyl sulfide	(CH_3_)_2_S	SC	-	3.50 ± 0.27	-	-	-	-	-
3	8.66	917	915	Ethyl acetate	C_4_H_8_O_2_	E	0.39 ± 0.06	-	-	-	-	-	1.41 ± 0.22
4	9.21	936	933	2-Methyl-butanal	C_5_H_10_O	CC	-	0.48 ± 0.02	-	-	-	-	-
5	9.33	940	949	3-Methyl-butanal	C_5_H_10_O	CC	-	0.46 ± 0.07	0.32 ± 0.05	-	-	-	-
6	10.44	973	975	2-Ethyl-furan	C_6_H_8_O	FC	-	0.41 ± 0.02	-	-	-	-	-
7	11.23	995	997	3-Pentanone	C_5_H_10_O	CC	-	0.82 ± 0.15	-	-	-	-	-
8	12.48	1024	1022	Methyl 2-methylbutanoate	C_6_H_12_O_2_	E	-	-	0.17 ± 0.03	-	-	-	-
9	12.79	1031	1034	1-Penten-3-one	C_5_H_8_O	CC	-	3.72 ± 0.51	-	-	-	-	-
10	13.36	1042	1046	α-Pinene	C_10_H_16_	M	-	-	0.19 ± 0.01	-	27.00 ± 1.83	0.11 ± 0.01	-
11	14.69	1068	1076	Sabinene	C_10_H_16_	M	-	-	-	-	0.20 ± 0.02	-	-
12	14.91	1072	1070	Camphene	C_10_H_16_	M	-	-	-	-	0.78 ± 0.03	0.07 ± 0.01	-
13	15.83	1088	1090	Hexanal	C_6_H_12_O	CC	1.17 ± 0.05	5.84 ± 0.27	0.13 ± 0.02	1.93 ± 0.12	-	0.06 ± 0.01	0.26 ± 0.02
14	16.96	1107	1110	β-Pinene	C_10_H_16_	M	-	-	0.25 ± 0.03	-	40.10 ± 3.49	0.08 ± 0.00	-
15	17.63	1120	1140	α-Phellandrene	C_10_H_16_	M	-	-	-	-	34.35 ± 5.40	-	-
16	17.86	1124	1127	2-Pentenal	C_5_H_8_O	CC	-	0.52 ± 0.02	-	-	-	-	-
17	19.06	1145	1147	3-Carene	C_10_H_16_	M	-	-	0.12 ± 0.01	-	259.59 ± 38.11	-	-
18	19.44	1151	1157	1-Penten-3-ol	C_5_H_10_O	A	-	2.00 ± 0.31	-	-	-	-	-
19	19.91	1159	1159	β-Myrcene	C_10_H_16_	M	-	-	0.21 ± 0.01	-	0.54 ± 0.04	0.98 ± 0.18	2.58 ± 0.15
20	20.81	1173	1176	Heptanal	C_7_H_14_O	CC	-	-	0.13 ± 0.02	-	-	-	-
21	21.68	1186	1180	(E)-2-Hexenal	C_6_H_10_O	CC	-	1.33 ± 0.25	-	-	-	-	-
22	21.94	1190	1187	Limonene	C_10_H_16_	M	-	-	0.26 ± 0.04	-	34.42 ± 1.49	0.42 ± 0.04	0.47 ± 0.08
23	22.39	1196	1198	Eucalyptol	C_10_H_18_O	M	-	34.75 ± 3.87	0.10 ± 0.02	-	-	0.20 ± 0.04	0.11 ± 0.01
24	22.41	1196	1193	(Z)-2-Hexenal	C_6_H_10_O	CC	-	-	-	-	1.54 ± 0.23	1.42 ± 0.05	1.49 ± 0.24
25	22.51	1198	1198	β-Phellandrene	C_10_H_16_	M	-	-	-	-	105.26 ± 3.03	-	0.14 ± 0.02
26	2281	1203	1209	3-Methyl-1-butanol	C_5_H_12_O	A	-	-	0.37 ± 0.02	-	-	-	-
27	22.92	1205	1222	Ethyl hexanoate	C_8_H_16_O_2_	E	-	-	-	-	-	0.07 ± 0.01	1.04 ± 0.17
28	24.67	1235	1242	γ-Terpinene	C_10_H_16_	M	-	-	-	-	1.74 ± 0.25	-	-
29	24.88	1238	1243	*Trans*-β-ocimene	C_10_H_16_	M	-	-	0.73 ± 0.10	-	1.68 ± 0.13	39.41 ± 1.76	1.56 ± 0.16
30	25.06	1241	1238	1-Pentanol	C_5_H_12_O	A	-	-	0.15 ± 0.01	-	-	-	-
31	25.49	1248	1253	3-Octanone	C_8_H_16_O	CC	-	3.28 ± 0.17	-	-	-	-	0.32 ± 0.04
32	25.83	1254	1257	p-Cymene	C_10_H_14_	M	-	0.58 ± 0.03	-	-	0.42 ± 0.02	-	-
33	26.77	1268	1271	α-Terpinolene	C_10_H_16_	M	-	-	-	-	0.57 ± 0.05	-	-
34	28.11	1288	1287	2-Penten-1-ol	C_5_H_10_O	A	-	1.55 ± 0.04	-	-	-	-	-
35	28.25	1290	1291	3-Hexen-1-ol acetate	C_8_H_14_O_2_	E	-	7.14 ± 0.53	-	-	-	-	7.73 ± 0.78
36	28.75	1297	1305	2-Hexen-1-ol acetate	C_8_H_14_O_2_	E	-	-	-	-	-	-	4.04 ± 0.49
37	29.07	1303	1327	Ethyl heptanoate	C_9_H_18_O_2_	E	-	-	-	-	-	0.07 ± 0.00	-
38	29.28	1306	1317	6-Methyl-5-hepten-2-one	C_8_H_14_O	CC	-	-	0.35 ± 0.04	-	0.19 ± 0.01	0.11 ± 0.01	0.65 ± 0.08
39	30.41	1326	1321	1-Hexanol	C_6_H_14_O	A	1.57 ± 0.23	26.93 ± 2.06	0.73 ± 0.10	-	2.70 ± 0.05	0.52 ± 0.02	1.85 ± 0.29
40	30.46	1327	1325	Allyl isothiocyanate	C_4_H_5_NS	SC	-	-	-	9.39 ± 1.63	-	-	-
41	30.57	1329	1331	(E)-3-Hexen-1-ol	C_6_H_12_O	A	-	0.74 ± 0.13	-	-	-	-	-
42	31.76	1349	1357	(Z)-3-Hexen-1-ol	C_6_H_12_O	A	-	59.38 ± 5.91	1.83 ± 0.17	-	1.47 ± 0.06	6.18 ± 0.02	-
43	31.82	1350	1351	*Trans*-alloocimene	C_10_H_16_	M	-	-	-	-	1.28 ± 0.19	-	-
44	31.84	1351	1361	(E)-2-Hexen-1-ol	C_6_H_12_O	A	-	-	-	1.13 ± 0.11	-	-	-
45	32.92	1368	1373	(E)-4-Hexen-1-ol	C_6_H_12_O	A	-	8.01 ± 0.89	0.21 ± 0.01	-	1.13 ± 0.01	0.17 ± 0.03	0.73 ± 0.11
46	33.43	1377	1374	Fenchone	C_10_H_16_O	M	-	-	-	-	0.27 ± 0.03	0.20 ± 0.02	-
47	34.95	1400	1429	Ethyl octanoate	C_10_H_20_O_2_	E	-	-	-	-	-	0.30 ± 0.02	-
48	35.42	1409	1420	1-Octen-3-ol	C_8_H_16_O	A	-	11.68 ± 2.01	0.28 ± 0.02	-	-	-	-
49	35.75	1416	1430	1-Heptanol	C_7_H_16_O	A	2.63 ± 0.21	-	0.36 ± 0.02	-	-	-	-
50	36.12	1422	1431	6-Methyl-5-hepten-2-ol	C_8_H_14_O	A	-	-	-	-	-	-	0.41 ± 0.03
51	36.52	1430	1416	3-Butenyl isothiocyanate	C_5_H_7_NS	SC	-	-	-	29.92 ± 1.74	-	0.34 ± 0.01	2.57 ± 0.15
52	37.11	1441	1442	Citronellal	C_10_H_18_O	M	-	-	-	-	-	-	0.24 ± 0.02
53	37.33	1445	1445	α-Cubebene	C_10_H_16_	S	-	-	-	-	10.93 ± 1.78	-	-
54	37.52	1448	1444	2-Ethyl-1-hexanol	C_8_H_18_O	A	-	-	-	-	-	-	0.36 ± 0.01
55	37.84	1454	1452	δ-Elemene	C_10_H_16_	S	-	-	-	-	11.59 ± 1.57	-	-
56	37.90	1455	1440	(Z)-3-Hexenyl 3-methylbutanoate	C_10_H_18_O_2_	E	-	-	-	-	-	0.37 ± 0.06	-
57	38.40	1464	1473	Cyclosativene	C_10_H_16_	S	-	-	-	-	9.15 ± 0.05	-	-
58	38.59	1467	1466	2-Hexenyl butanoate	C_10_H_18_O_2_	E	-	-	-	-	-	0.30 ± 0.05	-
59	38.84	1472	1477	Copaene	C_10_H_16_	S	-	-	-	-	10.63 ± 0.69	-	1.86 ± 0.31
60	39.12	1476	1495	β-Bourbonene	C_10_H_16_	S	-	-	-	-	9.27 ± 0.52	0.34 ± 0.03	-
61	39.58	1484	1491	Benzaldehyde	C_7_H_6_O	CC	-	-	-	-	-	1.42 ± 0.22	-
62	40.33	1497	1509	(E)-2-Nonenal	C_9_H_18_O	CC	-	-	-	-	-	1.58 ± 0.20	-
63	40.62	1502	1503	Ethyl nonanoate	C_10_H_20_O_2_	E	-	-	0.25 ± 0.04	-	-	1.02 ± 0.07	1.63 ± 0.29
64	40.89	1507	1512	Theaspirane B	C_10_H_16_O	S	-	-	-	-	-	-	1.71 ± 0.07
65	41.21	1514	1516	1-Octanol	C_8_H_18_O	A	-	-	2.24 ± 0.30	-	-	-	0.34 ± 0.06
66	41.29	1515	1535	Cyperene	C_10_H_16_	S	-	-	-	-	19.41 ± 3.14	-	-
67	41.61	1521	1519	β-Cubebene	C_10_H_16_	S	-	-	-	-	12.66 ± 3.54	-	-
68	42.92	1546	1548	(E,Z)-2,6-Nonadienal	C_10_H_18_O	CC	-	-	-	-	-	0.80 ± 0.11	-
69	43.99	1566	1553	Linalyl formate	C_10_H_10_O_2_	E	-	-	-	-	337.61 ± 26.25	-	-
70	45.00	1584	1581	β-Caryophyllene	C_10_H_16_	S	-	-	-	-	1599.59 ± 213.65	1.88 ± 0.32	0.23 ± 0.01
71	45.72	1596	1589	Aromandrene	C_10_H_16_	S	-	-	-	-	1.90 ± 0.20	-	-
72	45.82	1598	1605	Benzeneacetaldehyde	C_8_H_8_O	CC	-	-	-	-	-	1.39 ± 0.10	-
73	46.68	1616	1635	δ-Cadinene	C_10_H_16_	S	-	-	-	-	5.89 ± 0.70	-	-
74	47.00	1622	1622	Humelene	C_10_H_16_	S	-	-	-	-	10.32 ± 0.57	-	-
75	47.07	1624	1634	Ethyl benzoate	C_11_H_10_O_2_	E	-	-	-	-	-	0.26 ± 0.02	-
76	47.79	1638	1638	β-Ciclocitral	C_10_H_10_O_2_	M	-	-	-	-	-	-	3.79 ± 0.65
77	47.84	1639	1648	β-Farnesene	C_10_H_16_	S	-	-	-	-	2.52 ± 0.34	-	-
78	48.47	1652	1644	α-Caryophyllene	C_10_H_16_	S	-	-	-	-	144.7 ± 14.5	-	0.64 ± 0.04
79	49.17	1665	1685	(E)-3-Nonen-1-ol	C_9_H_18_O	A	-	-	-	-	-	0.12 ± 0.02	-
80	49.41	1670	1670	α-Muurolene	C_10_H_16_	S	-	-	-	-	47.51 ± 3.15	-	-
81	50.13	1684	1685	Germacrene D	C_10_H_16_	S	-	-	-	-	51.10 ± 0.90	1.21 ± 0.12	-
82	50.30	1687	1692	α-Citral	C_10_H_16_O	S	-	-	-	-	-	0.12 ± 0.02	31.10 ± 1.93
83	50.53	1691	1672	Epizonarene	C_10_H_16_	CC	-	-	-	-	231.91 ± 27.06	0.53 ± 0.08	-
84	50.83	1697	1694	β-Bisabolene	C_10_H_16_	S	-	-	-	-	411.64 ± 66.82	0.10 ± 0.01	-
85	51.39	1708	1711	Geranyl acetate	C_12_H_18_O_2_	S	-	-	-	-	-	-	8.74 ± 0.54
86	51.47	1710	1716	Bicyclogermacrene	C_10_H_16_	S	-	-	-	-	14.45 ± 2.34	-	-
87	51.82	1717	1706	α-Farnesene	C_10_H_16_	S	-	-	-	-	25.49 ± 2.61	0.48 ± 0.07	-
88	52.67	1734	1710	δ-Amorphene	C_10_H_16_	S	-	-	-	-	71.05 ± 10.91	-	-
89	52.80	1736	1730	Methyl salicylate	C_8_H_8_O_3_	E	-	-	-	-	-	65.81 ± 5.92	-
90	53.26	1745	1725	γ-Muurolene	C_10_H_16_	M	-	-	-	-	-	-	13.69 ± 1.77
91	53.81	1756	1738	δ-Selinene	C_10_H_16_	S	-	-	-	-	6.61 ± 0.87	-	-
92	54.14	1762	1780	2-Phenylethyl acetate	C_10_H_12_O_2_	E	-	-	-	-	-	-	19.80 ± 1.32
93	54.19	1763	1780	Ethyl salicylate	C_10_H_12_O_3_	E	-	-	-	-	-	23.19 ± 1.89	-
94	54.21	1764	1753	β-Muurolene	C_10_H_16_	S	-	-	-	-	23.10 ± 1.60	-	-
95	55.11	1781	1797	Geraniol	C_10_H_18_O	M	-	-	-	-	65.26 ± 2.07	-	371.74 ± 55.28
96	55.93	1796	1812	β-Amorphene	C_10_H_16_	S	-	-	-	-	6.67 ± 0.21	-	-
97	58.37	1847	1858	Phenylethyl alcohol	C_8_H_10_O	A	-	-	-	22.42 ± 0.72	-	1.35 ± 0.23	49.19 ± 8.47
98	61.37	1902	1914	α-Calacorene	C_10_H_16_	S	-	-	-	-	1.25 ± 0.24	-	-
99	62.51	1908	1928	Caryophyllene oxide	C_10_H_16_O	S	-	-	-	-	5.36 ± 0.22	-	-
100	64.09	2016	2008	Nerolidol	C_10_H_18_O	S	-	-	-	-	1.85 ± 0.15	-	-
101	72.07	2154	2167	T-Cadinol	C_10_H_18_O	S	-	-	-	-	1.17 ± 0.09	-	-
102	74.62	2165	-	Cariophylladienol I	C_10_H_16_O	S	-	-	-	-	1.08 ± 0.08	-	-

CC: Carbonyl compounds; SC: Sulfur compounds; E: Esters; FC: Furanic compounds; M: Monoterpenoids; A: Alcohols; S: Sesquiterpenoids; -: Not detected; ^a^ RT: Retention time; ^b^ Kovats index relative *n*-alkanes (C_8_ to C_20_) on a SUPELCOWAX^®^ 10 capillary column; ^c^ Kovats index relative reported in the literature for equivalent capillary column [26,27,28]; ^d^ MF: Molecular formula; ^e^ Relative area: VOM area/3-octanol area.

**Table 2:** Potential bioactive effects reported in the literature of some of the most active VOMs identified in the investigated edible flowers.

Peak nº	VOMs	Edible Flowers							Potential Bioactive Effects
		*Begonia* spp.	*Borago officinalis*	*Anthirrinum majus*	*Lobularia maritima*	*Acmella oleracea*	*Viola tricolor*	*Rosa* spp.	
3	Ethyl acetate	x						x	Antimicrobial
10	α-Pinene			x		x	x		Antidiabetic, antifungal, antioxidant, antiproliferative, antitumor, cytotoxic
11	Sabinene					x			Antibacterial, antifungal
12	Camphene					x	x		Antimicrobial, antioxidant
13	Hexanal	x	x	x	x		x	x	Antimicrobial
14	β-Pinene			x		x	x		Antitumor, anti-inflammatory, antimicrobial, antioxidant, antineoplastic, chemoprotective
17	3-Carene			x		x			Antimicrobial, antioxidant, anticancer
19	β-Myrcene			x		x	x	x	Analgesic, anti-inflammatory, antibiotic, anticancer, antioxidant
22	Limonene			x		x	x	x	Antimutagenic, antitumor, antioxidant, antimicrobial, antiproliferative, chemoprotective
23	Eucalyptol		x	x			x	x	Anti-inflammatory, antioxidative, antihyperglycemic, antimicrobial, antihypertensive, anti-tumoral, antinociceptive, antipyretic, analgesic
25	β-Phellandrene					x		x	Antibacterial, antifungal, anticancer, antidiabetic, antioxidant, analgesic, antiviral, anti-inflammatory
28	γ-Terpinene					x			Anti-inflammatory, antioxidant
32	p-Cymene		x			x			Antibacterial, anti-inflammatory, antifungal, antioxidant, cytotoxic
33	α-Terpinolene					x			Antioxidant
35	1-Octanol			x				x	Antifungal
39	1-Hexanol	x	x	x		x	x	x	Antifungal
40	Allyl isothiocyanate				x				Anticancer, antibacterial, antifungal, anti-inflammatory, antioxidant
48	1-Octen-3-ol		x	x					Antioxidant
49	1-Heptanol	x		x					Antifungal
51	3-Butenyl isothiocyanate				x		x	x	Cytotoxic, anticancer
70	β-Caryophyllene					x	x	x	Antibacterial, antidiabetic, antioxidant, antiproliferative, cytotoxic
84	β-Bisabolene					x			Cytotoxic, anti-inflammatory, antibacterial
89	Methyl salicylate						x		Anti-inflammatory, analgesic, antipyretic, antifungal
90	γ-Muurolene							x	Anti-inflammatory, antimicrobial, antioxidant, cytotoxic
95	Geraniol					x		x	Chemopreventive activity, antimutagenic, anti-inflammatory
97	Phenylethyl alcohol				x		x	x	Antimicrobial, antioxidant, antienzymatic
99	Caryophyllene oxide					x			Antibacterial, antioxidant, antiproliferative, cytotoxic, analgesic

## Data Availability

The original contributions presented in this study are included in the article/Supplementary Material. Further inquiries can be directed to the corresponding author(s).

## Supplementary Materials

The following supporting information can be downloaded at https://www.mdpi.com/article/10.3390/molecules30081799/s1: Figure S1: Typical total ion chromatograms (TIC) of edible flowers obtained using HS-SPME/GC-MS; Table S1: Number of VOMs identified and the total relative area found in the investigated flowers.

## Abbreviations

The following abbreviations are used in this manuscript:

VOM	Volatile organic metabolite
HS-SPME	Headspace solid-phase microextraction
GC-MS	Gas chromatography coupled to mass spectrometry
RT	Retention time
KI	Kovats index
MF	Molecular formule
SD	Standard deviations
CC	Carbonyl compounds
SC	Sulphur compounds
E	Esters
FC	Furanic compounds
M	Monoterpenoids
A	Alcohols
S	Sesquiterpenoids
PCA	Principal component analysis
PLS-DA	Partial least squares-discriminant analysis
PC	Principal component
VIP	Variable importance in the projection
HCA	Hierarchical cluster analysis
IS	Internal standard
DVB/CAR/PDMS	Divinylbenzene/carboxen/polydimethylsiloxane
FS	Full scan
q-MS	Quadrupole mass spectrometry detector
EI	Electron impact ionization
NIST	National Institute of Standards and Technology
