# Supplementary material for: Edible Flowers in Modern Gastronomy: A Study of Their Volatilomic Fingerprint and Potential Health Benefits
Source: Molecules. 2025 Apr 17;30(8):1799. doi: 10.3390/molecules30081799 (PMC12029428; doi:10.3390/molecules30081799)
Supplement: Supplementary file 1 [file molecules-30-01799-s001.zip › molecules-3566795-supplementary.pdf]

Edible Flowers in Modern Gastronomy: A Study of Their Volatilomic Fingerprint and Potential Health Benefits

Begoña Fernández-Pintor [1,2], Rosa Perestelo [3], Sonia Morante-Zarcero [1], Isabel Sierra [1,2,*] and José S. Câmara [3,4,*]

[1] Departamento de Tecnología Química e Ambiental, Escuela Superior de Ciencias Experimentales y Tecnología, Universidad Rey Juan Carlos, C/Tulipán s/n, 28933 Móstoles, Madrid, Spain; begona.fernandez@urjc.es (B.F.-P.); sonia.morante@urjc.es (S.M.-Z.)
[2] Instituto de Tecnologías para la Sostenibilidad, Universidad Rey Juan Carlos, C/Tulipán s/n, 28933 Móstoles, Madrid, Spain
[3] CQM—Centro de Química da Madeira, Universidade da Madeira, Campus da Penteada, 9020-105 Funchal, Portugal; rmp@staff.uma.pt
[4] Departamento de Química, Facultade de Ciències Exatas e Engenharia, Universidade da Madeira, Campus da Penteada, 9020-105 Funchal, Portugal
* Correspondence: isabel.sierra@urjc.es (I.S.); jsc@staff.uma.pt (J.S.C.)

a)

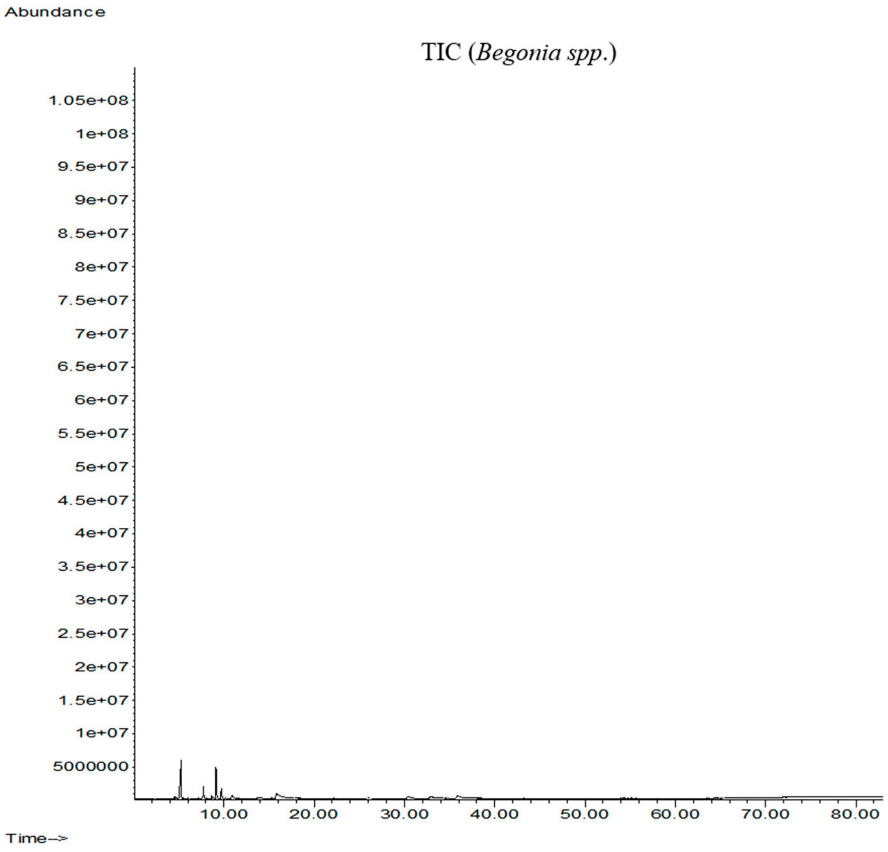
Abundance
TIC (*Begonia spp.*)
1.05e+08
1e+08
9.5e+07
9e+07
8.5e+07
8e+07
7.5e+07
7e+07
6.5e+07
6e+07
5.5e+07
5e+07
4.5e+07
4e+07
3.5e+07
3e+07
2.5e+07
2e+07
1.5e+07
1e+07
5000000
10.00
20.00
30.00
40.00
50.00
60.00
70.00
80.00
Time-->

b)

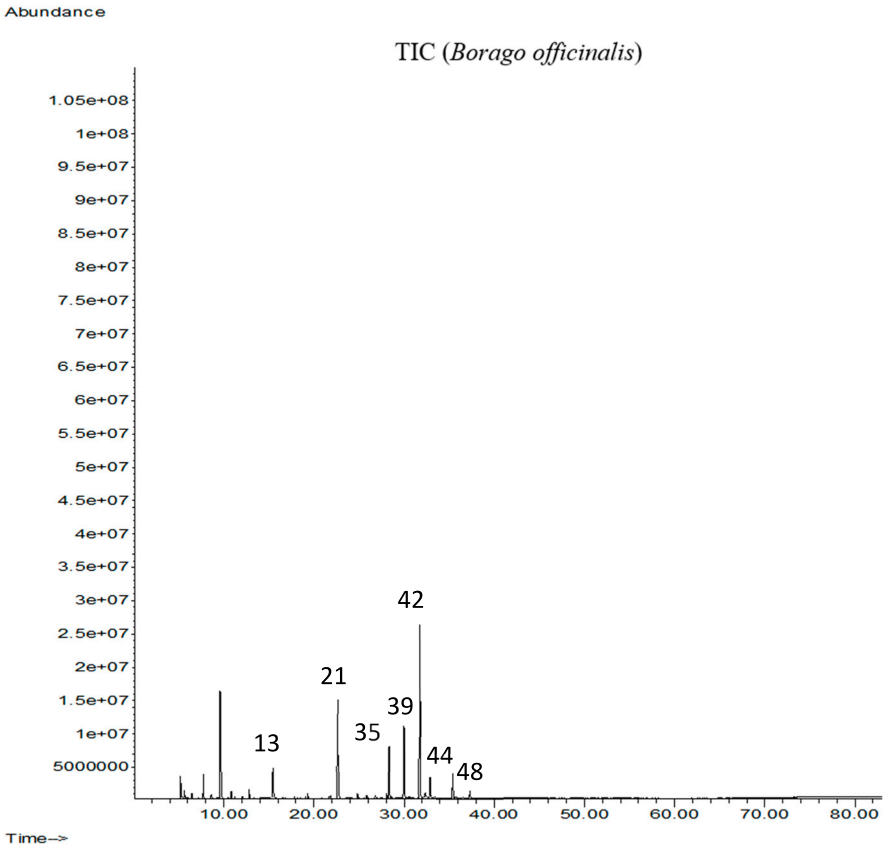
Abundance
TIC (*Borago officinalis*)
1.05e+08
1e+08
9.5e+07
9e+07
8.5e+07
8e+07
7.5e+07
7e+07
6.5e+07
6e+07
5.5e+07
5e+07
4.5e+07
4e+07
3.5e+07
3e+07
2.5e+07
2e+07
1.5e+07
1e+07
5000000
42
21
39
35
13
44
48
10.00
20.00
30.00
40.00
50.00
60.00
70.00
80.00
Time-->

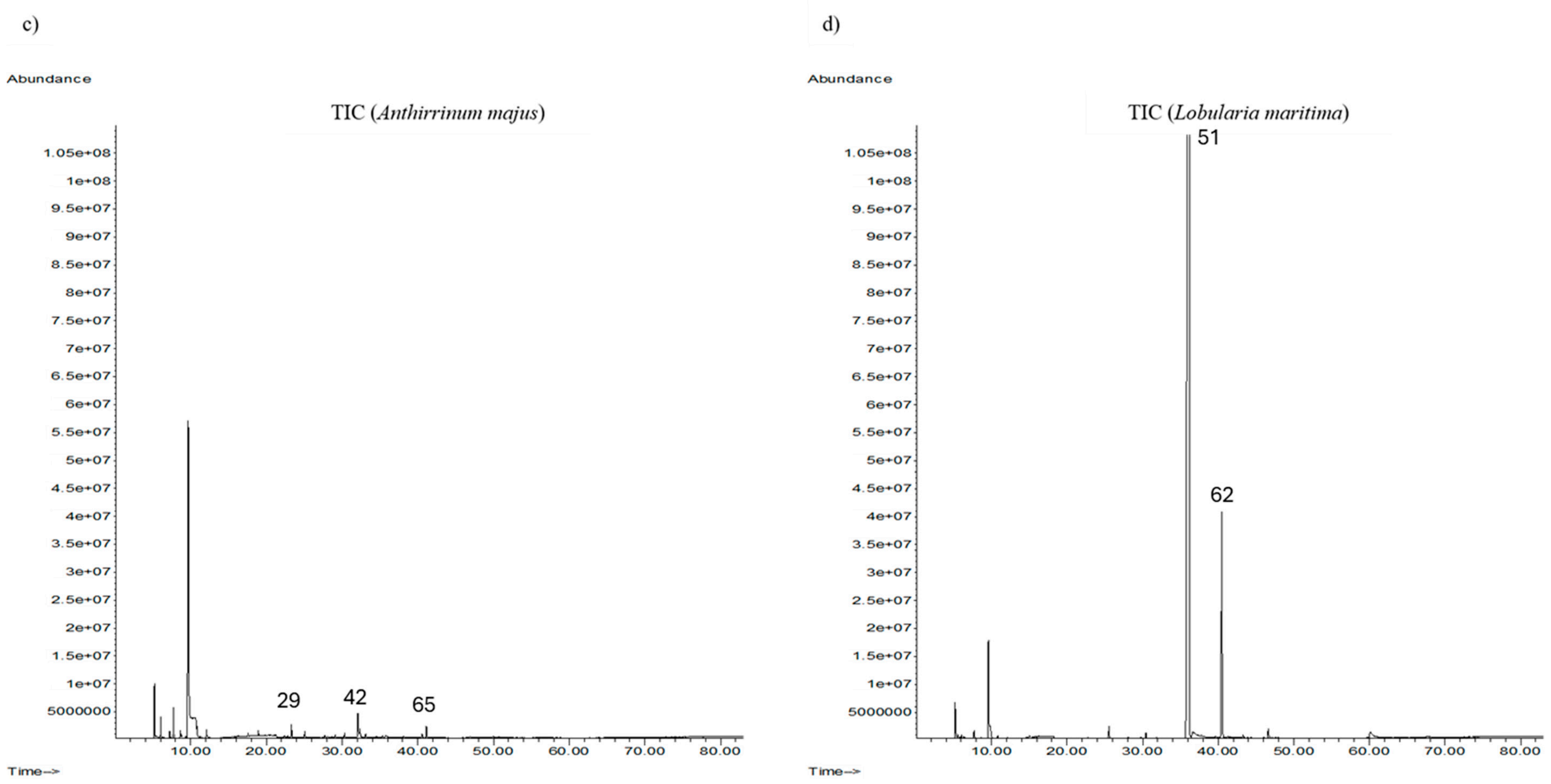
c)
Abundance
TIC (*Anthirrinum majus*)
1.05e+08
1e+08
9.5e+07
9e+07
8.5e+07
8e+07
7.5e+07
7e+07
6.5e+07
6e+07
5.5e+07
5e+07
4.5e+07
4e+07
3.5e+07
3e+07
2.5e+07
2e+07
1.5e+07
1e+07
5000000
29
42
65
10.00 20.00 30.00 40.00 50.00 60.00 70.00 80.00
Time-->
d)
Abundance
TIC (*Lobularia maritima*)
51
62
1.05e+08
1e+08
9.5e+07
9e+07
8.5e+07
8e+07
7.5e+07
7e+07
6.5e+07
6e+07
5.5e+07
5e+07
4.5e+07
4e+07
3.5e+07
3e+07
2.5e+07
2e+07
1.5e+07
1e+07
5000000
10.00 20.00 30.00 40.00 50.00 60.00 70.00 80.00
Time-->

e)

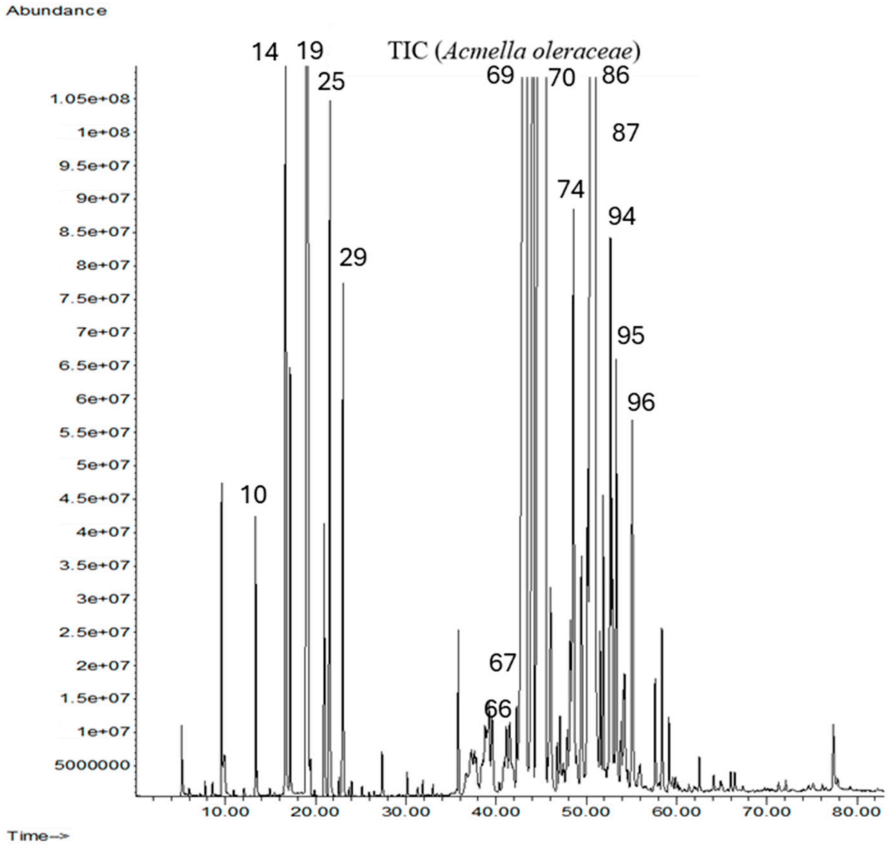
Abundance
TIC (Acmella oleraceae)
14
19
25
29
10
69
70
86
87
74
94
95
96
67
66
1.05e+08
1e+08
9.5e+07
9e+07
8.5e+07
8e+07
7.5e+07
7e+07
6.5e+07
6e+07
5.5e+07
5e+07
4.5e+07
4e+07
3.5e+07
3e+07
2.5e+07
2e+07
1.5e+07
1e+07
5000000
10.00 20.00 30.00 40.00 50.00 60.00 70.00 80.00
Time-->

f)

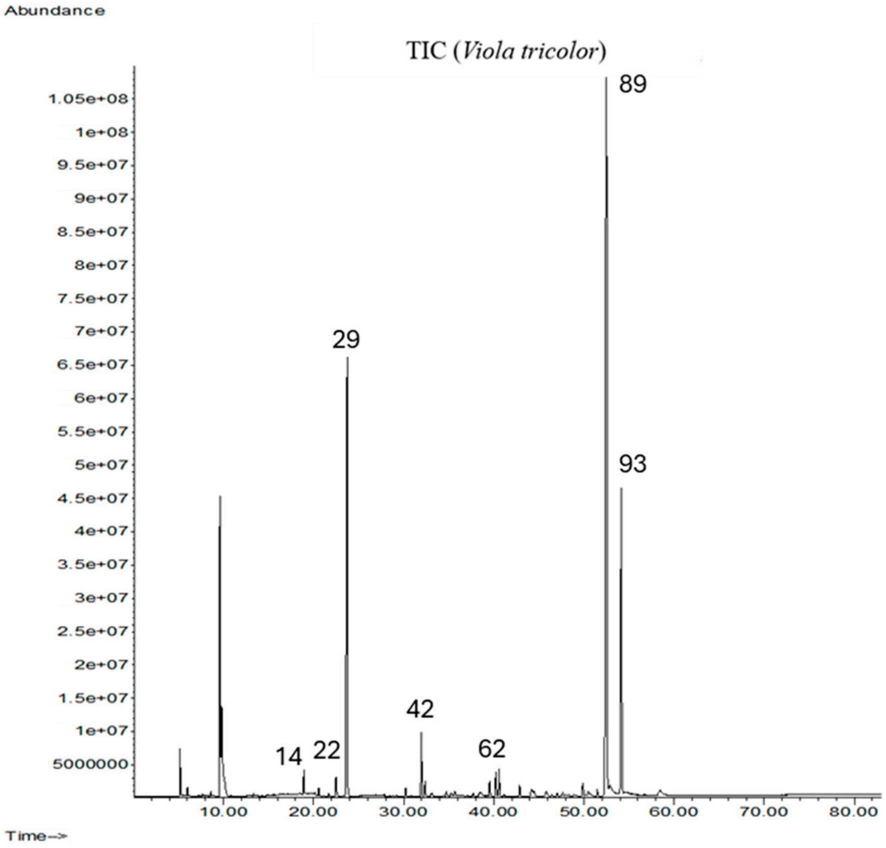
Abundance
TIC (Viola tricolor)
89
29
93
42
14
22
62
1.05e+08
1e+08
9.5e+07
9e+07
8.5e+07
8e+07
7.5e+07
7e+07
6.5e+07
6e+07
5.5e+07
5e+07
4.5e+07
4e+07
3.5e+07
3e+07
2.5e+07
2e+07
1.5e+07
1e+07
5000000
10.00 20.00 30.00 40.00 50.00 60.00 70.00 80.00
Time-->

g)

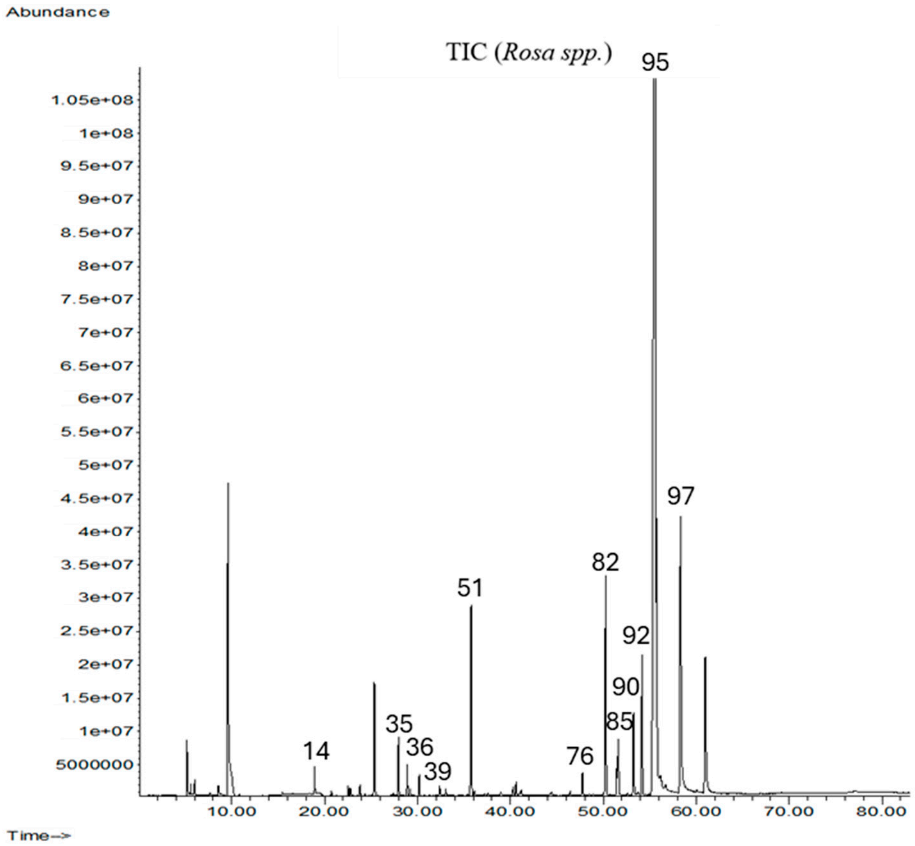

Figure S1. Typical total ion chromatograms (TIC) of edible flowers obtained using HS-SPME/GC-MS. Peak numbers (Table 1): 10: α-Pinene; 13: Hexanal; 14: β-Pinene; 19: β-Myrcene; 21: (E)-2-Hexenal; 22: Limonene; 25: β-Phellandrene; 29: *Trans*-β-ocimene; 35: 3-Hexen-1-ol acetate; 36: 2-Hexen-1-ol acetate; 39: 1-Hexanol; 42: (Z)-3-Hexen-1-ol; 44: (E)-2-Hexen-1-ol; 48: 1-Octen-3-ol; 51: 3-Butenyl isothiocyanate; 62: Benzaldehyde; 65: 1-Octanol; 66: Cyperene; 67: β-Cubebene; 69: Linalyl formate; 70:

β-Caryophyllene; 74: Humelene; 76: β-Ciclocitral; 82: α-Citral; 85: Geranyl acetate; 86: Bicyclogermacrene; 87: α-Farnesene; 89: Methyl salicylate; 90: γ-Muurolene; 92: 2-Phenylethyl acetate; 93: Ethyl salicylate; 94: β-Muurolene; 95: Geraniol; 96: β-Amorphene; 97: Phenylethyl alcohol

Table S1. Number of VOMs identified and the total relative area found in the investigated flowers.

	Begonia ssp.	*Borago officinalis*	*Anthirrinum majus*	*Lobularia maritima*	*Acmella oleracea*	*Viola tricolor*	*Rosa spp.*
Nº VOMs	4	20	22	6	50	38	33
Total relative area (µg/L)	6	173	10	44	3782	153	905
